# Malaria surveillance amongst pregnant women attending antenatal care in private hospitals in Onitsha metropolis, South Eastern Nigeria

**Published:** 2022-06-21

**Authors:** Moses N. Ikegbunam, Chibuzo Uba, Judith Flügge, Harrison Abone, Dorothy Ezeagwuna, Simeon Ushie, Charles Esimone

**Affiliations:** 1 Department of Pharmaceutical Microbiology and Biotechnology, Nnamdi Azikiwe University, Awka, Nigeria; 2 Molecular Research Foundation for Students and Scientists, Nnamdi Azikiwe University, Awka, Nigeria; 3 Institute for Tropical Medicine, Tübingen, Germany; 4 Department of Parasitology and Entomology, Nnamdi Azikiwe University, Awka, Nigeria; 5 Department of Medical Microbiology and Parasitology, Nnamdi Azikiwe University, Awka, Nigeria

## Abstract

**Background:**

Recent reports suggest that pregnant women living in holoendemic regions of sub-Sahara Africa die in great numbers annually due to malaria disease resulting from their higher susceptibility, reduced immunity and demographic associated factors. This work investigated the prevalence of *Plasmodium falciparum* in pregnant women attending antenatal care (ANC) in selected private hospitals in Onitsha metropolis South East Nigeria.

**Methods:**

Venous blood samples were collected from 270 pregnant women during ANC visits between October 2016 and December 2017. A questionnaire was used to collect demographic data, gestational age, knowledge of malaria and preventive measures while clinical presentations and symptoms were extracted from the physician’s clerking form. Laboratory diagnosis was done using microscopy. The effect of the demographic variables and other associated factors on prevalence and parasite densities was studied using Chi-square and ANOVA tests.

**Results:**

The overall *P. falciparum* prevalence was 42.6%. Prevalence varied with the maternal age, gestational age, preventive measures adopted by the pregnant women and clinical presentations. 27.8 % of the infected women were highly parasitized (>5000 parasites/μl); 67% had a moderate parasite density (1,000-4,999 parasites/μl) and 5.2% showed a low parasite density (1-999 parasites/μl). We observed that 35.2%, 30%, 18.9% and 5.2% of the study cohorts preferred and used treated bed nets, insecticides, windows and door screening and non-treated bed nets respectively as malaria preventive measures. 5.9% did not use any protection.

**Conclusions:**

The findings of this study revealed high prevalence of malaria among pregnant women living in Onitsha metropolis with high mean parasite densities despite strong adherence to use of sulphadoxine-pyrimethamine (SP) for intermittent preventive treatment in pregnancy (IPTp) and other malaria preventive measures.

## Introduction

Malaria disease is considered a major public health concern among pregnant women especially in tropical and subtropical areas of the world and in sub-Sahara Africa, where malaria vectors are commonly found. Malaria in pregnancy results in adverse pregnancy outcomes, such as spontaneous abortion, neonatal death, and low birth weight. Chronic anemia, due to malaria may also affect a child’s growth and intellectual development. Pregnant women are particularly vulnerable to malaria infection as a result of reduced immunity [1].

In malaria-endemic regions of Africa, pregnancies are threatened by malaria. Recently, prevalence of exposure to malaria during pregnancy was highest in Central Africa (40%), closely followed by West Africa (39%), while East and Southern Africa had a reported exposure prevalence of 24% [2]. Pregnant women residing in areas with stable malaria transmission are at a greater risk of serious malaria. Pregnant women in such places are at a greater risk of serious malaria than non-pregnant women, and therefore face a higher mortality rate due to malaria [3,4].

Nigeria contributes more malaria than other endemic regions in Africa, with 27% and 23% of global malaria cases and global malaria deaths respectively [2]. Following these reports and other available data in the literature, malaria is holoendemic in many parts of Nigeria with transmission occurring throughout the year [1]. These increases the vulnerability of pregnant women, since there is an increased likelihood of infection due to perennial transmission. The transmission of malaria is affected by many environmental and socio-economic factors, including sanitation, housing, literacy and poverty, among others [5]. Furthermore, WHO has linked malaria to poverty, because it is believed to more severely affect the less privileged in society that cannot afford to pay for malaria treatment [6]. WHO has once listed Onitsha in Anambra State as one of the most polluted cities and like many other endemic regions facing the same challenge, it is exposed to high transmission of malaria parasites [7].

Due to the fact that pregnant women have greater vulnerability and decreased immunity, many malaria control programmes are concerned about their safety especially those residing in malaria-endemic nations, so there is need for regular updated information on the prevalence of malaria among pregnant women, especially in endemic areas. A review of the existing literature over the last decade revealed only a few publications that documented the prevalence of malaria among pregnant women in Onitsha [8,9,10]. Therefore, this study assessed the prevalence of *P. falciparum* among pregnant women attending ANC in selected private hospitals in Onitsha metropolis as well as the impact of control strategies on their respective parasite densities.

## Materials and Methods

### Study area

Onitsha is located in Anambra State and lies between Latitudes 06005120.8911N and 06013126.47311N and Longitude 06045120.60411E and 06052110.57311E. It is situated on the eastern side of the River Niger and covers approximately 50 square kilometers [11]. The average annual rainfall in Onitsha is approximately 1,850 millimeter with varying annual temperature ranging from 23°C to 37°C [11]. Onitsha is a business area having the biggest market in West Africa.

### Study design

This was a cross-sectional study with a sample size of 270 pregnant women (calculated using Cochran equation described by Anto *et al.* [12]) scheduled for ANC visits.

Participants with clinical diagnosis of fever (temperature >37.5°C) and/or a history of fever, and headache as reported by the physician, were included. Socio-demographic data were captured using a pre-tested questionnaire. Ethical clearance was obtained from the UNTH Ethical Board (Reference No - approval number : NHREC/05/01/2008B-FWA00002458-IRB00002323). Written informed consent was obtained from all study participants.

### Sample collection and microscopy

Between October 2016 and December 2017, about 2 ml of venous blood was collected into anti-coagulant containers from pregnant women that attended the ANC. Samples were analysed and examined by a WHO-certified microscopist using a standard procedure [13]. Blood specimens collected in EDTA bottles were used to make thick and thin films on a microscope slide. The films were stained with 10% Giemsa stain (pH 7.2) for 10 min and then viewed under an oil immersion objective of a microscope (at 1,000x magnification). Parasitaemia was calculated using the formula recommended by WHO [14] as follows:

Parasite Density = [(Number of Parasites counted)/(WBC counted)] x Assumed WBC count.

Samples were labeled negative if no parasites were detected after observing 200 fields of a thick blood film.

### Data presentation and analysis

All data were presented and calculated using SPSS statistical package (version 22). The effect of the demographic variables and other associated factors on prevalence and parasite densities was studied using Chi-square and ANOVA tests, respectively, with a significance level of p≤0.05 and confidence level of 95%.

## Results

### Demographic characteristics

The demographic characteristics of the 270 women as captured by the questionnaire are shown in Table 1. Most of the participants (37.8%) fell into the age group of 27-32 yrs. The study population had a mean age of 27.41±5.56 yrs. The majority of participants (40.7%) were in their 2^nd^ trimester and had enjoyed secondary education (47.8%).

**Table 1.** Prevalence of P. falciparum in pregnant women (n=270) with respect to demographic variables, prophylaxis, vector control measures and clinical presentations.

	Variables		Microscopy		
		n	Negative (%)	Positive (%)	P-value
Age group (years)	15-20	23	9 (39.1)	14(60.9)	
	21-26	101	61 (60.4)	40 (39.6)	
	27-32	102	62 (60.8)	40 (39.2)	
	33-38	35	18 (51.4)	17 (48.6)	
	>38	9	5 (55.6)	4 (44.4)	
	**Total**	**270**	**155 (57.4)**	**115 (42.6)**	**0.341**
Gestation age (months)	1-3	82	42 (51.2)	40 (48.8)	
	4-6	110	63 (57.3)	47 (42.7)	
	7-9	78	50 (64.1)	28 (35.9)	
	**Total**	**270**	**155 (57.4)**	**115 (42.6)**	**0.257**
Education	Primary	24	9 (37.5)	15 (62.5)	
	Secondary	129	72 (55.8)	57 (44.2)	
	Tertiary	115	73 (63.5)	42 (36.5)	
	**Total**	**268**	**154 (57.5)**	**114 (42.5)**	**0.056**
Antimalaria drug used	Amodiaquine	1	1(100)	0 (0)	
	AL*	30	15 (50)	15 (50)	
	Quinine	18	8 (42.1)	11 (57.9)	
	SP**	113	58(51.3)	55(48.7)	
	None	68	51(75)	17(25)	
	**Total**	**181**	**109 (60.2)**	**72 (39.8)**	**0.009**
Vector control measures	Window/door screens	51	29 (56.9)	22 (43.1)	
	Treated nets	95	55 (57.9)	40 (42.1)	
	Untreated nets	14	5 (35.7)	9 (64.3)	
	Insecticides	81	49 (60.5)	32 (39.5)	
	Insecticides and others	8	5 (62.5)	3 (37.5)	
	No protection	16	10 (62.5)	6 (37.5)	
	**Total**	**265**	**153 (57.1)**	**112 (42.3)**	**0.658**
Clinical symptoms	Headache	65	49 (75.4)	16 (24.6)	
	Headache and fever	93	20 (21.5)	73 (78.5)	
	Fever	18	7 (38.9)	11 (61.1)	
	**Total**	**269**	**154**	**115 (42.8)**	**≤0.001**

* AL = Artemether-lumefantrine; ** SP = Sulphadoxine- pyrimethamine

### Malaria prevention

Nearly half of the participants (48.9%) had used SP for prophylaxis against malaria infection prior to participation in the study; nearly a third (29.4%) had not received any prophylaxis. Some participants used quinine (8%), amodiaquine (0.4%) or artemether-lumefantrine (AL) (13%), which are not usually recommended for use during pregnancy. The use of insecticide-treated bed nets and insecticides (indoor residual spraying) was higher (35.8% and 30.6%, respectively) than any other vector control measure adopted by the women.

### Clinical signs and symptoms

Clinical presentations as recorded on the ANC cards by doctors and nurses were extracted and are shown in Table 1. Asymptomatic women represented 34.8 % of the total population. Additionally, 34.4 % of the women presented with headache and fever. Fewer women reported only headache (24.1%) or only fever (6.7%).

### *P. falciparum* prevalence

Microscopy confirmed 115 of the blood samples as positive, giving an overall prevalence of 42.6% (Table 1). Parasite densities were calculated for the positive slides and categorized, showing that 27.8% had high parasitaemias (>5,000 parasites/μl), 67% of moderate (1,000-4,999 parasites/μl) and 5.2% had a low parasite parasitaemia (1-999 parasites/μl). *P. falciparum* prevalence with respect to age, gestational age, prophylaxis using SP, vector control measures and clinical symptoms is shown in Table 1 while the effect of these associated risk factors on parasite density is presented in Table 2.

**Table 2.** Effect of demographic variables, gestational age, preventive measures, prophylaxis and clinical symptoms on mean parasite densities (MPD±SD) in pregnant women (n=270).

	**Variables**	**n**	**MPD±SD**	**P-value**
Age group (years)	15-20	15	6053 ± 7664	
	21-26	40	4291 ± 2436	
	27-32	40	3814 ± 3089	
	33-38	17	4078 ±1396	
	>38	4	2419 ±1396	
	**Total**	**115**		**0.33**
Gestation age (months)	1-3	40	4463 ± 3162	
	4-6	47	4283 ± 4827	
	7-9	28	3861 ± 2594	
	**Total**	**115**		**0.81**
Education	Primary	15	3517 ± 2490	
	Secondary	57	5068 ± 5068	
	Tertiary	43	3402 ± 2286	
	**Total**	**115**		**0.68**
Vector control measures	Window/door screens	22	4508 ± 4579	
	Treated nets	40	4314 ± 4474	
	Untreated nets	9	5617 ± 3221	
	Insecticides	32	4082 ± 2853	
	Insecticides and others	3	4152 ± 2749	
	No Protection	6	2501 ± 2270	
	**Total**	**112**		**0.77**
Antimalaria drug use	AL*	15	3427 ± 1871	
	Quinine	11	5353 ± 5626	
	SP**	55	4212 ± 2893	
	None	17	6342 ± 6269	
	**Total**	**98**		**0.14**
Clinical symptoms	Asymptomatic	15	1847 ± 1318	
	Headache	16	3764 ± 1949	
	Headache and Fever	73	4931 ± 4301	
	Fever	11	3644 ± 3284	
	**Total**	**115**		**0.03**

* AL: Artemether-lumefantrine; ** SP: Sulphadoxine- pyrimethamine

Prevalence decreased with age. The highest prevalence (60.9%) was observed in the youngest age cohort (15-20 yrs). In contrast, 27-32 yrs group had the lowest prevalence (39.2%; p>0.05) (Table 1). Mean parasite density for the 15-20 yrs cohort (6053±7664 parasite/μl) was higher than those for the other age groups. Mean parasite densities decreased with age although these observed differences were not significant (Table 2).

The frequency of parasite occurrence was high (48.20%) among pregnant women in their 1^st^ trimester (1-3 months) than among those in 2^nd^ (4-6 months) and 3^rd^ trimesters (42.70% and 35.90% respectively) (Table 1) at p>0.05. Women in their 1^st^ trimester had a higher mean parasite density of 4463±3162 parasite/μl, compared to those in 2^nd^ and 3^rd^ trimesters who had a mean parasite density (parasite/μl) of 4283±4827 and 3861±2594 respectively (p>0.05) (Table 2).

The prevalence of *P. falciparum* infection decreased with increasing level of education among pregnant women (Table 1). Those who only had primary education had the highest prevalence rate (62.5%) compared with those who had secondary (44.20%) and tertiary education (36.50%) at p>0.05. The mean parasite density was higher in those women whose highest level of education was a secondary education (5068±5068 parasite/μl) compared to those with primary education (3517±2490 parasite/μl) and tertiary education (3402±2286 parasite/μl) at p>0.05 (Table 2).

The prevalence rate of *P. falciparum* was higher among pregnant women who used quinine (57.9%) than those who had used AL (50%) and SP (48.7) at p<0.05 (Table 1). However, the mean parasite density was higher among those who had not received prophylaxis (6342±6269 parasites/μl) compared to those who have used SP (4212±2893 parasites/μl) and AL (3427±1871 parasites/μl) at p>0.05 (Table 2).

It was further observed that the prevalence rate of *P. falciparum* was higher among pregnant women who used non-treated bed nets (64.30%) than among those who used windows and door nets (43.10%), treated bed nets (42.10%) and insecticides (IRS) (39.50%). The observed difference was not statistically significant at p≤0.05.

The distribution of parasite density with respect to the type of malaria prevention method used by the pregnant women participating in the study is shown in Table 2. Mean parasite density was higher in those who used non-treated bed nets (5617±3221 parasites/μl) compared to others who used windows and door nets (4508±4579 parasites/μl), treated bed nets (4314±4474 parasites/μl), insecticides (IRS) (4082±2853 parasites/μl) or insecticides and others (4152±2749 parasites/μl) at p>0.05.

The prevalence rate of *P. falciparum* among pregnant women with respect to the clinical symptoms was higher among those with headache and fever (78.50%) than in asymptomatic individuals who recorded only 16.10% of the *P. falciparum* infections. Furthermore, the mean parasite density was higher in those who indicated headache and fever (4931±4301parasites/μl) compared to those who indicated headache only (3764±1949 parasites/μl) or fever only (3644±3284 parasites/μl). Among the asymptomatic individuals, the mean parasite density was considerably low (1847±1319 parasites/μl). The observed effect was statistically significant at p≤0.05 (Table 2).

## Discussion

Malaria remains a serious public health challenge across Africa and Nigeria in particular. It is detrimental to the health of pregnant women and their unborn child [15]. In this study, we determined *P. falciparum* prevalence among pregnant women who visited private hospitals for ANC in Onitsha Anambra state, Nigeria.

Malaria prevalence was found to be high. This may be attributed to a several factors ranging from increased attractiveness to mosquitoes [16] to immunological and physiological changes required for the survival of the fetus [15]. Also, environmental factors such as blockage of drainage systems, large population of people living in poor housing as well as high environmental pollution posing a serious challenge to people living in Onitsha metropolis as documented by WHO [7] can create a good breeding habitat for mosquitoes and hence the increased prevalence rate observed in this study. This finding is consistent with some recent reports of high malaria prevalence rates among pregnant women attending ANC units across Nigerian states [17].

A high prevalence rate of *P. falciparum* and parasite density was observed among young pregnant women compared to older pregnant women. Even though this was not statistically significant, many studies have attributed this observation to lower immunity of younger pregnant women against malaria [15,18]. Through frequent and repeated malaria infections, older pregnant women produce more antibodies to malaria parasites and therefore gain higher immunity than younger pregnant women protecting them against generation of high parasite densities below detectable levels and clinical symptoms [18]. This corresponds to previous investigations which demonstrated that malaria is more common among younger pregnant women [17,19]. This study revealed that the prevalence rate of *P. falciparum* and parasite density decreased nonsignificantly with increase in gestational age. It is expected that parasite load should be higher in the third trimester compared to first and second trimester, due to progressive immunomodulation in pregnancy, a state needed for fetal survival wherein the mother’s systemic immune reaction is altered towards the humoral arm, significantly noticed in the third trimester than the first [20]. This deviation could be due to improved understanding and use of malaria control strategies like treated bed nets and alternative intermittent preventive treatment with SP among the study participants. This observation has been reported for other similar studies in sub-Saharan Africa including South Eastern Nigeria [21-24]. The prevalence of *P. falciparum* infection and parasite density was higher among pregnant women with primary school education and secondary school education than those with tertiary education. However, the finding was not statistically significant. The use of antimalarial drugs significantly affected the prevalence but not the distribution of parasite density among the study participants. This finding, like other previous reports, suggests that the use of intermittent preventive treatment with IPTp-SP or AL as indicated in this study were apparently effective at malaria prevention and treatment as it resulted to a reduced parasite density among the pregnant women who used them [25,26].

Also, *P. falciparum* prevalence and density was higher among those who used non-treated bednets compared to the prevalence rate and density obtained among those who used treated bednets and other preventive measures even though no statistically significant difference was observed. Majority of the participants preferred the use of insecticidetreated bednets as a prevention measure more than other preventive measures, and this may have contributed to a lower observed malaria prevalence. In Nigeria, insecticide treated bednets have been reported to be an effective measure of preventing malaria. Ownership and use of long-lasting insecticidal nets (LLIN) are proven interventions adopted by Roll Back Malaria (RBM) partners in Nigeria to stem the high incidence of malaria [27].

However, both prevalence and mean parasite density was higher in symptomatic pregnant women than among the asymptomatic individuals. This suggests that the symptoms and clinical presentation of malaria cases during pregnancy is signifcantly dependent on parasite load and can be useful for early detection. It further approves the use of clinical presentation as first line diagnosis for malaria in pregnant women and immediate treatment of patients with fever, headache, or headache and fever with antimalarial drugs, where laboratory screening and confirmation is not readily available. Treatment based on clinical presentation in pregnant women may help prevent complications associated with malaria in pregnancy as well as prevent potentially fatal disease among those living in rural settings.

Finally, a high mean parasite density was recorded contrary to the thought that pregnant women usually have reduced number of parasites in the blood circulation due to proliferation of parasites in the placenta [28,29]. High parasite densities as obtained in this study could be associated to accumulation of multiple clones and genetic diversity of *P. falciparum* parasites which gives room for parasite immune evasion including drug resistance especially in areas of high transmission [30-32]. Studies in other parts of Nigeria have shown high genetic diversity in *P. falciparum* isolates [33-36] suggesting the possibility of high transmission, drug resistance and subsequently a high parasite density among people living in this region.

## Conclusions

The prevalence of malaria among pregnant women living in the Onitsha is considerably high. There is a strong adherence to the use of SP for IPTp among pregnant women attending ANC in selected hospitals. Also, there is a strong adherence to the use of treated bednets, and window and door screening as malaria prevention measures. Despite strong adherence to SP for IPTp and other malaria preventive measures, the findings of this study revealed a high mean parasite density among the (especially younger) study cohort. Therefore, efforts should be made towards studying and understanding the molecular basis for the reported high prevalence and parasite density among pregnant women. Malaria elimination programmes should focus on strategies that will help pregnant women in poor and highly dirty environments to understand the risk of exposure to malaria infection as well as provide access to malaria prevention measures including ITNs and LLINs so as to reduce the malaria burden in this population.
